# Migration Behavior of Low-Density Particles in Lab-on-a-Disc Devices: Effect of Walls

**DOI:** 10.3390/mi12091032

**Published:** 2021-08-28

**Authors:** Vyacheslav R. Misko, Agata Kryj, Aude-Muriel Tamandjo Ngansop, Sogol Yazdani, Matthieu Briet, Namanya Basinda, Humphrey D. Mazigo, Wim De Malsche

**Affiliations:** 1 µFlow Group, Department of Bioengineering Sciences, Vrije Universiteit Brussel, 1050 Brussels, Belgium; veaceslav.misco@vub.be (V.R.M.); Agata.Kryj@vub.be (A.K.); aude-muriel.tamandjo.ngansop@vub.be (A.-M.T.N.); sogol.yazdani@vub.be (S.Y.); matthieu.briet@vub.be (M.B.); namanya.samson.basinda@vub.be (N.B.); 2 Department of Medical Parasitology and Entomology, School of Medicine, Catholic University of Health and Allied Sciences, Mwanza 33000, Tanzania; humphreymazigo@gmail.com

**Keywords:** particle separation, parasite egg identification and quantification, diagnostic microfluidic device, extreme point-of-care

## Abstract

The effect of the lateral walls of a Lab-On-a-Disc device on the dynamics of a model system of particles with a density lower than that of the solvent (modelling parasites eggs) is analyzed theoretically and experimentally. In the absence of lateral walls, a particle always moves in the direction of the centrifugal force, while its trajectory is deflected in the tangential direction by the inertial Coriolis and Euler forces. Lateral walls, depending on the angle forming with the radial direction, can guide the particle either in the same or in the opposite direction to the centrifugal force, thus resulting in unusual particle trajectories including zig-zag or backwards particle motion. The effect is pronounced in the case of short operation times when the acceleration of the angular rotation, and thus the Euler force, is considerable. The predicted unusual motion is demonstrated by numerically solving the equation of motion in the presence of lateral walls and verified in the experiment with particles of density lower than that of the solvent. Our analysis is useful for design and operational considerations of Lab-On-a-Disc devices aiming for or involving (bio)particle handling.

## 1. Introduction

A Lab-On-a-Disc (LOD) device is a centrifugal microfluidic device that can be referred to as a subclass of integrated Lab-on-a-Chip (LOC) platforms [1,2,3,4]. In addition to the advantages of a LOC platform such as portability, the use of small amounts of materials and reagents, faster reaction times, and programmability [2], the LOD platform employs pseudo-forces generated during the rotation of the device: centrifugal force, the Coriolis force, and the angular acceleration-generated Euler force [3].

A wide range of applications including clinical chemistry, immunoassay, cell analysis [5,6], and nucleic acid tests [7,8,9], could be demonstrated on a spinning disc [10]. Typical examples of applications of these LOD platforms are sample-to-answer systems for biomedical point-of-care and global diagnostics [11], liquid handling automation for the life sciences (e.g., concentration/purification and amplification of DNA/RNA from a range of bio-samples), process analytical techniques and cell line development for biopharma as well as monitoring the environment, infrastructure, industrial processes and agrifood [12,13].

The LOD platform has been applied for detection and molecular analysis of pathogens [14] such as, e.g., *Salmonella*, a major food-borne pathogen [15,16]. Thus, a centrifugal microfluidic device was developed [15], which integrated the three main steps of pathogen detection, DNA extraction, and isothermal recombinase polymerase amplification (RPA) and detection, onto a single disc. Very recently, possibilities of employing LOD platforms for the detection of Covid-19 have been discussed in the literature [17].

Recently, a LOD device was proposed [18] for the detection of soil-transmitted helminths (STH) which are intestinal worms that infect humans and are spread through contaminated soil [19,20]. The device represents a centrifugal microfluidic platform based on centrifugation and flotation which isolates and collects eggs within an imaging zone using saturated sodium chloride as flotation solution. The main advantage of this device (for other diagnostics methods, see: [21,22,23,24,25]) is that it provides quick diagnostics where needed (point-of-care testing) and requires small amounts of sample and materials. This device provided fast and efficient operation and an image of a packed monolayer of eggs collected within a single imaging zone. To enable the separation and to improve the purification efficiency, secondary flotation and size-based separation mechanisms were implemented in the platform. To provide a complete analysis platform, a bench-top imaging setup was coupled to the centrifugation unit, designed and constructed. This platform was equipped with a high-resolution imaging unit and light source and was ready for wireless data transfer. The distinct feature of this system is that a parasite egg monolayer can be formed by restricting the chamber height of the imaging zone to the size of a single egg (as low as 60 µm). The consumer camera that we have used can in future be replaced by a smartphone. This would allow for a further reduction of the instrument cost and can allow for a more widespread implementation of the technique. The platform was successfully tested in Ethiopia, Tanzania, Uganda and Kenya on infected human and animal samples for evaluation of the developed technology (unpublished results).

Several studies on particle centrifugation are focused on the optimization of the efficiency of the devices intended for performing specific tasks, while the mechanism of centrifugation is assumed to be well-established. Indeed, it is the well-understood inertial (pseudo-)force, called centrifugal force, that causes the radial motion of particles in a centrifuge and in this way, e.g., separates various types of particles depending on their density. Thus, at first sight, the mechanism of centrifugation is very simple. However, a more detailed look at the dynamics of particles in a chamber of a centrifuge device reveals a rather complex motion pattern due to the presence of other forces, in addition to the centrifugal force, exerted on a particle in a centrifuge device.

Here we analyze—experimentally and theoretically—the effects related to these additional forces, including the inertial Coriolis and Euler forces, and the effect of lateral walls of the chamber on centrifugation of particles. First, we consider centrifugation of heavy particles (density particles higher than of the surrounding liquid, *ρ_p_* > *ρ_f_*) where the above effects are intuitively more transparent and can thus be easier understood, and then we analyze these effects for particles with the density lower than that of the surrounding fluid, representative of parasite eggs immersed in a solution, to understand complex motion patterns and their impact on the efficiency of centrifugation, with the ultimate goal of optimization of the LOD design.

## 2. Effect of Lateral Walls

### 2.1. Governing Forces

The motion of a particle of mass *m* in a centrifuge device obeys Newton’s equation of motion:

(1) $$m\vec{a}=\underset{i}{\sum }{\vec{F}}_{i},$$

where $\vec{a}$ is the acceleration, and $\underset{i}{\sum }{\vec{F}}_{i}$ is the sum of all the forces exerted on the particle. Rotation with angular velocity ω results in effective inertial forces, also called pseudo-forces, acting on the particle even in the absence of external forces:

(2) $$m\vec{a}=-m\frac{d\vec{\omega }}{dt}\times \vec{r}-2m\vec{\omega }\times \vec{v}-m\vec{\omega }\times \left(\vec{\omega }\times \vec{r}\right)+\underset{i}{\sum }{\vec{F}}_{i}$$

Here, $-m\frac{d\vec{\omega }}{dt}\times \vec{r}$ is the Euler force, which is due to the angular acceleration, i.e., essential during the initial phase of rotation, before the centrifuge reaches the maximum rotation speed ω, and also during slowing down at the end of rotation. Note that the Euler force is directed normal to the radius-vector of the particle position, $\vec{r}$, in the plane of the rotation. The term $-2m\vec{\omega }\times \vec{v}$ represents the Coriolis force that depends on the velocity of the particle and not on the particle position. Its direction is determined by the direction of the motion. For example, if a particle moves outwards from the center of the disc, the Coriolis force deflects the motion in the direction opposite to the rotation. Finally, the term $-m\vec{\omega }\times \left(\vec{\omega }\times \vec{r}\right)$ expresses the centrifugal force that only depends on the particle position and the angular velocity ω, and is directed along the radius. In most cases, the centrifugal force is considered as the main governing force leading to the centrifugation effect [26]. Indeed, only the centrifugal force is directed along the radius and thus ultimately causes the motion of particles from the center to the periphery or in the opposite direction. However, even the Euler force and the Coriolis force that are normal to the radius of the rotation and to the direction of the motion, respectively, can lead to striking effects such as zig-zag trajectories and even backwards movements of particles, as demonstrated below. These unusual effects result from the interaction of the moving particles with the lateral walls of the centrifuge chamber.

The above inertial forces in Equation (2), which are mentioned in the literature on centrifugal devices (see, e.g., [3]), come purely from the rotation. They result from the formal transformation of the equation of motion, Equation (1), to a frame rotating with angular velocity ω.

Next, a particle of density *ρ_p_* immersed in a fluid of density *ρ_f_* experiences the Archimedes buoyancy force:

(3) $$F_{b,\;A}=V_{p}g\left(\rho_{p}-\rho_{f}\right)$$

where g and *V_p_* are the gravitational acceleration and the particle volume, respectively.

In a rotating frame, in addition to the Archimedes buoyancy, valid for an inertial motion in the gravity field, one should take into account also the rotating buoyancy [10] related to the accelerations resulted from the rotation. Since all the inertial forces in Equation (2) are due to the accelerations induced by the rotation, all of them should be modified for particles immersed in a fluid. Thus, the modified centrifugal force taking into account the rotating buoyancy becomes:

(4) $$F_{c,br}=-V_{p}\left(\rho_{p}-\rho_{f}\right)\vec{\omega }\times \left(\vec{\omega }\times \vec{r}\right)$$

The modified Euler and Coriolis forces are, correspondingly:

(5) $$F_{E,br}=-V_{p}\left(\rho_{p}-\rho_{f}\right)\frac{d\vec{\omega }}{dt}\times \vec{r}$$

and:

(6) $$F_{C,br}=-V_{p}\left(\rho_{p}-\rho_{f}\right)\vec{\omega }\times \vec{v}$$

In addition to the Archimedes buoyancy (3) and the inertial forces (4) to (6), the Stokes drag force:

(7) $$F_{d}=-6\eta \pi r_{p}v$$

is exerted on a particle of radius *r_p_* moving with velocity v in the fluid, as well as the forces due to the friction with the chamber walls.

### 2.2. Effect of Lateral Walls

To distinguish effects resulting from particle–wall interactions from other possible effects, let us first consider a heavy particle.

**Figure 1:**
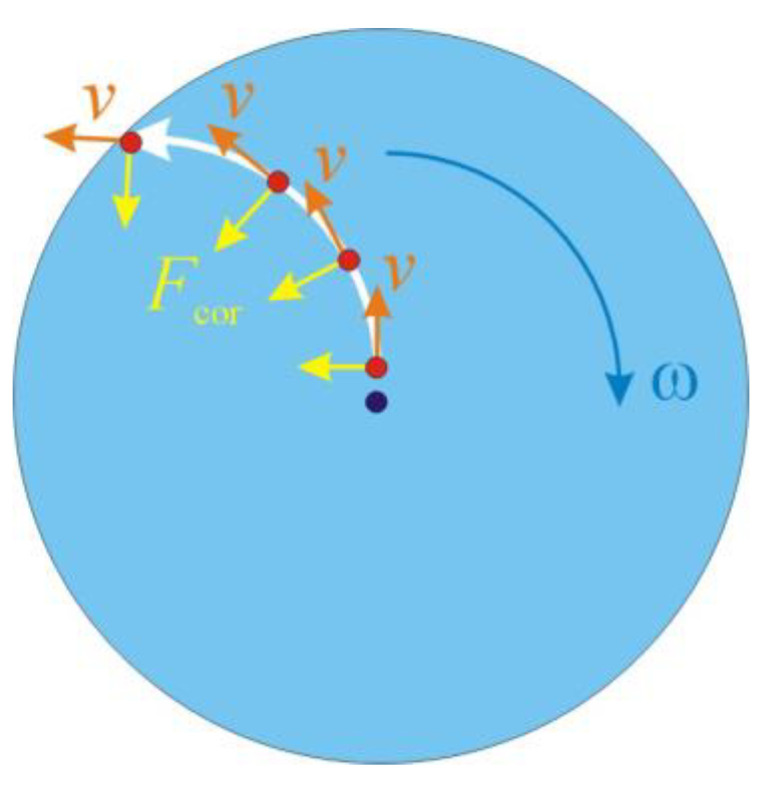
Trajectory of a particle moving from the center of a rotating disc towards its edge, in a disc rotating with angular velocity ω. The direction of the velocity, *v*, is deflected from the radial direction of the centrifugal force by the Coriolis force *F_cor_*.

In the absence of lateral walls, the particle trajectory is represented by the curve shown in Figure 1.

If we now imagine a rectangular chamber placed on the disc then, depending on the width of the disc, the direction of the particle motion and of the Coriolis force at the wall will differ (as shown in Figures S1a,b in the Supplementary Materials). For a narrow chamber, the direction of motion will be close to parallel to the wall (Figure S1a), while for a wider channel the particle would reach the chamber wall at a larger angle γ (Figure S1b). This means that the projection of the particle velocity on the chamber wall, *v_w_* = *v*∙cos(γ), will be small. The direction of motion of the particle along the wall in this case is determined by the balance between the projections of the centrifugal force (towards the edge) and the Coriolis force (towards the center) on the direction of the wall, during the collision with the wall (Figure S1c). Note that the Coriolis force results in this case in a transient effect, related to the inertial motion of the particle after the collision, which is rapidly damped by the Stokes drag and the friction with the wall. However, the Euler force due to eventual radial acceleration of the disc is not evanescent and can compensate and even overcome the projection of the centrifugation force. In the case that it does not overcome the radial migration, the motion still persists in the direction towards the edge of the disc: the particle drifts along the disc with a smaller velocity than it would do in the absence of the wall. Note that this drift velocity could be very small, due to additional effects of velocity slowing down near the wall [27]. Wall imperfections can lead to sticking of the particle at the wall.

If the trajectory of a moving particle deviates more strongly towards the wall (due to a faster rotation or angular acceleration), then the particle arrives at the wall at a larger angle, close to normal. In this case the projection of the Coriolis and Euler forces are larger than the projection of the centrifugal force on the direction of the wall (Figure S2a), and the resulting velocity of the particle after the collision with the wall is directed inwards, i.e., opposite to the centrifugal force. In other words, the interaction with a chamber wall can lead to a change in the direction of the centrifugation velocity to the opposite. After the collision, the particle drifts along the wall, not to the direction of the centrifugation but towards the center of the disc.

However, such a drift towards the center, contrarily to the drift in the direction of the centrifugation (Figure S1d) is not stable. When moving towards the center, the Coriolis force exerted on the particle also changes the direction to the opposite, and the particle moves away from the wall (Figure S2b). In this way, the particle becomes “free” again and then it follows a typical trajectory for a free particle until it hits the wall again.

It is clear that the above scenario can lead to unusual motion patterns such as zig-zag-type trajectories, when a particle moves towards the wall and repeatedly collides with the wall but still drifts towards the edge of the disc. Under other circumstances, if the rotation is fast enough or when the disc experiences sufficient angular acceleration, a particle can even display backwards motion, which, however, is not stable and can result in a rather complex trajectory consisting of repeated forwards and backwards movements and eventually zig-zag movements between the opposite lateral walls of the chamber.

**Figure 2:**
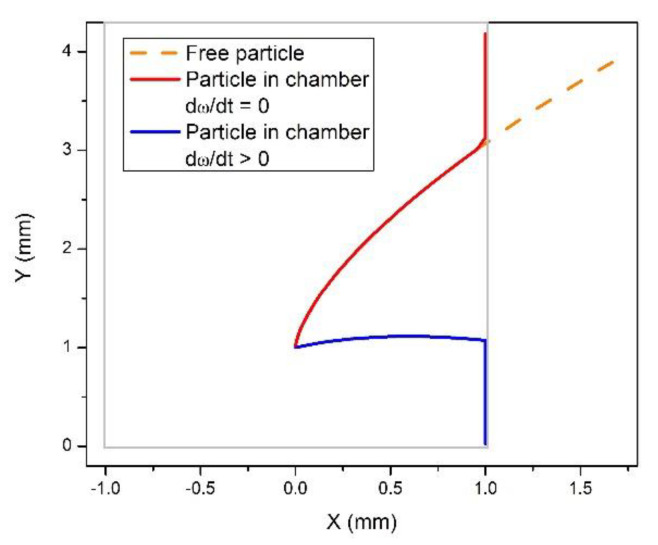
Calculated trajectories of a particle (*d_p_ =* 123 μm, *ρ_p_* = 1.05 g/mL, *ρ_f_* = 1 g/mL) in a rotating disc. The orange dashed line shows a trajectory of a free particle in a disc rotating around the point (0,0) in the counterclockwise direction with angular velocity ω = 800 rpm. A trajectory of the same particle in a rectangular chamber of 2 mm width (empty box) is modified: after the particle reaches the lateral wall of the chamber, it drifts under the action of the centrifugal force (red line). When an additional Euler force due to the angular acceleration (assuming linear increase of ω from 0 to 800 rpm in 0.5 s) is exerted on the particle, it moves backwards after reaching the wall (blue line).

To support this qualitative analysis, we solved the equation of motion, Equation (2), numerically, for model parameters, in a rotating frame in a rectangular chamber. Typical trajectories of particles are shown in Figure 2. A trajectory of a free particle, in the absence of lateral walls, is presented by a curve (orange dashed line in Figure 2) describing the motion when the distance from the center of rotation monotonically increases in time. In the presence of a lateral wall, a particle under the same rotation velocity reaches the wall and then drifts along the wall towards the periphery, being driven by the centrifugal force (red line). However, when a nonzero angular acceleration is present (as during the initial phase of the rotation) and the additional Euler force is exerted on the particle opposite to the direction of the rotation, the trajectory changes, and after reaching the wall the particle starts moving towards the center of rotation, i.e., opposite to the direction of the centrifugal force. This supports the above analysis predicting the possibility of backwards motion of particles in a centrifuge device (cp. Figure S2b).

### 2.3. Particles with Density Lower Than That of the Fluid

The above analysis revealed unusual behavior of particles in a centrifuge chamber of rectangular shape, for the case of particles with density larger than that of the fluid, *ρ_p_* > *ρ_f_*, where the predicted effects (such as a zig-zag and backwards movement) can be easily understood. The situation with “light” particles, i.e., those with density smaller than that of the fluid, *ρ_p_* < *ρ_f_*, is less intuitively clear. Indeed, in this case the particles can be considered as “bubbles” in a fluid. Contrarily to the situation described above, this is now the heavier fluid that experiences stronger inertial forces than the particles themselves. This means that now, e.g., during the initial phase of rotation characterized by the angular acceleration, the fluid will be affected by the Euler force stronger than the particles and, as a result, the fluid will move in the opposite direction to the angular acceleration, and the particles will move in the direction of the acceleration. The same is applied to other inertial forces: they act on the higher density fluid, and the lower density (and thus lighter) particles are excluded by the fluid via the pressure exerted on the particles from the fluid. Thus, the mechanism of motion of particles lighter than the fluid (*ρ_p_* < *ρ_f_*) is qualitatively different from that of “heavy” particles with *ρ_p_* > *ρ_f_*—their motion is mediated by the surrounding fluid.

However, the theoretical formalism, as formulated above in Equations (4)–(7), remains unchanged and applicable also for particles with *ρ_p_* < *ρ_f_*, same as the Archimedes buoyancy (3). Because of the condition *ρ_p_* < *ρ_f_*, the direction of the forces in Equations (3)–(7) changes to the opposite.

**Figure 3:**
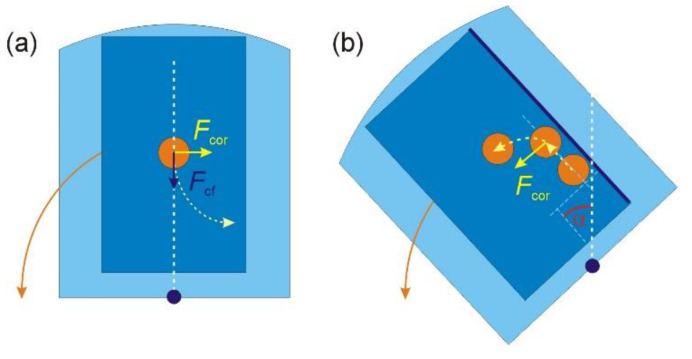
The motion of a particle with density smaller than that of the liquid, *ρ_p_* < *ρ_f_*, in a centrifuge chamber rotating counterclockwise: (**a**) the trajectory of a free particle moving towards the center under the action of the centrifugal force deviates opposite to the direction of rotation; (**b**) the backward motion of the particle for an angle *α* exceeding a critical value: *α > α_c_* (cp. Figure S2b).

The trajectory of a particle that moves towards the center under the action of the centrifuge force is deviated opposite to the direction of rotation (Figure 3a), under the action of the Coriolis force (that would normally lead to the deviation in the direction of rotation for a particle with *ρ_p_* > *ρ_f_*). Then, repeating the above discussion, valid for the case of *ρ_p_* > *ρ_f_*, for particles with *ρ_p_* < *ρ_f_*, we arrive at the situation shown in Figure 3b: for a large enough angle between the radial direction and the lateral wall of the chamber, *α > α_c_*, the particle executes a backward motion along the wall, opposite to the action of the centrifugal force, and then it detaches from the wall, due to the Coriolis force, and moves in the inner region of the chamber. After that, it becomes free and repeats the motion in a way as shown in Figure 3a, resulting in a zig-zag trajectory or in a trajectory containing backwards movements.

**Figure 4:**
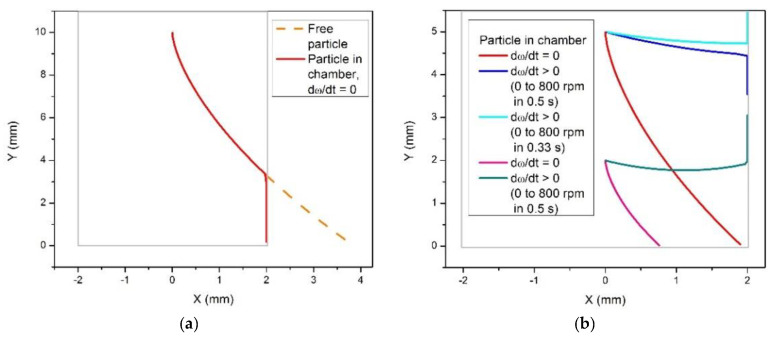
Calculated trajectories of a particle lighter than the fluid (*d_p_ =* 60 μm, *ρ_p_* = 1.05 g/mL, *ρ_f_* = 1.075 g/mL) in a rotating disc. (**a**) The orange dashed line shows a trajectory of a free particle in a disc rotating around the point (0,0) in the counterclockwise direction with angular velocity ω = 800 rpm. The red line shows the trajectory of a particle in a rectangular chamber of 4 mm width (the grey empty box). After reaching the lateral wall of the chamber, the particle keeps moving in the direction of the centrifugal force. (**b**) The red line shows the trajectory of a particle in the absence of the Euler force. The particle starts moving from the initial position (*x* = 0, *y* = 5 mm) and reaches the bottom of the chamber. When the Euler force (when ω linearly increases from 0 to 800 rpm in 0.5 s) is exerted on the particle, it achieves the wall and then moves along the wall in the direction of the centrifugal force (the blue line). A stronger Euler force (ω increases from 0 to 800 rpm in 0.33 s) leads to the backward motion (the light blue line). When the trajectory of the particle starts at a closer position to the center of rotation (*x* = 0, *y* = 2 mm), a weaker Euler force (ω = 0 to 800 rpm in 0.5 s) is sufficient for the backward motion (the green line).

**Figure 5:**
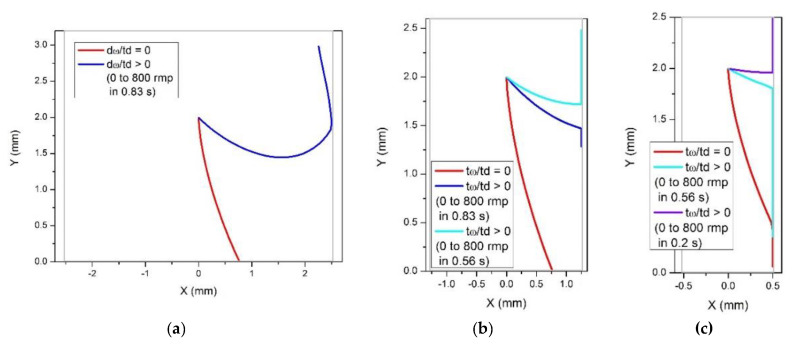
Calculated trajectories of light particles (using the same parameters as in Figure 4) in a rotating disc, in chambers of varying width. (**a**) Chamber width *w* = 5 mm. The red line shows the trajectory of a particle moving in a disc rotating with a constant angular velocity ω = 800 rpm. A weak angular acceleration (see the legend) is sufficient to cause a backward motion. (**b**) The same angular acceleration (the blue line) appears to be insufficient for a backward motion in a narrower chamber, *w* = 2.5 mm. The effect is achieved for a stronger Euler force (the light blue line). (**c**) In a very narrow chamber, *w* = 1 mm, even in the presence of rather strong angular accelerations the particle drifts towards the center of rotation. To turn the motion back, a very strong Euler force is required (see the legend).

The dynamics of light particles, i.e., particles with the density smaller than that of the fluid, has been further analyzed in numerical calculations. The results are presented in Figure 4 and Figure 5.

As predicted and illustrated in Figure 3a, a light particle moves towards the center of rotation under the action of the centrifugal force, and its trajectory is deviated in the direction opposite to the angular rotation (Figure 4a). To cause a backward motion, an angular acceleration (resulted in the Euler force) should be applied as illustrated in Figure 4b. The Euler force needed to flip the direction of the particle near the wall of the chamber depends on the distance of the particle from the center of rotation. Thus, to cause a backward motion of a particle at the distance *y* = 5 mm from the center of rotation, a rather strong angular acceleration is required, ω = 0 to 800 rpm in 0.33 s. For a particle positioned closer to the center of rotation, *y* = 2 mm, a smaller angular acceleration, ω = 0 to 800 rpm in 0.5 s, is sufficient to cause a backward motion. Therefore, the effect of eventual backward movements of centrifugated particles in chambers manifests itself preferably in the vicinity to the center of rotation.

Next, we analyze the effect of chamber width on the backward movements near the chamber boundary. Figure 5 presents results for three various chamber widths: *w* = 5 mm, 2.5 mm and 1 mm, for a particle initially positioned at (*x* = 0, *y* = 2 mm). In the case of a wide chamber, *w* = 5 mm (Figure 5a), a relatively small angular acceleration, ω = 0 to 800 rpm in 0.83 s, causes a backward motion of the particle after the collision with the boundary. Note that in this case the particle detaches from the wall and moves in the interior of the chamber as predicted and illustrated in Figure 3b. This motion is caused by the Coriolis force, which in this case appears to be strong enough to overcome the effect of the relatively weak Euler force. For narrower chambers, as shown in Figure 5b,c, stronger angular acceleration is required to turn the motion of the particle to the direction opposite to the centrifugal force. These observations confirm the above prediction that the possibility of a backward motion, i.e., opposite to the centrifugation force, of a particle in a centrifuge chamber depends on the angle between the radial direction and the direction of the wall. Our analysis suggests that the effect can be easier avoided for smaller angles that can be achieved (*i*) for particles situated further away from the center of rotation or (*ii*) in narrow chambers.

## 3. Experiment

**Figure 6:**
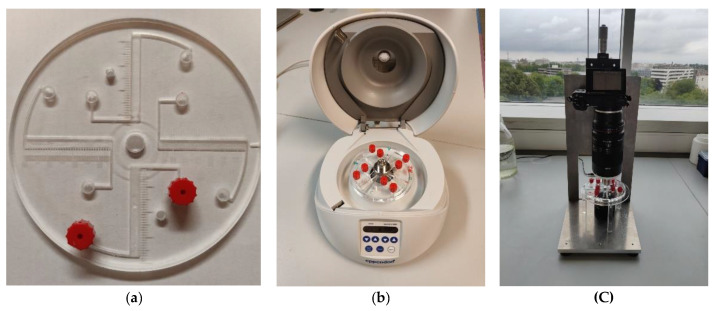
(**a**) LOD platform with four rectangular chambers. The channel width is 4 mm, the channel length is 35 mm, and the pitch of the scale bars is 1 mm. (**b**) The LOD platform in the centrifuge (Eppendorf^®^ Centrifuge MiniSpin G). (**c**) The photo imaging system: a camera with macro lens.

To analyze the effect of lateral walls on the dynamics of particles in a LOD-type device, we employed a LOD platform with rectangular chambers (Figure 6a). The injection chamber was placed further away from the flotation chamber to allow for the observation and validation of egg presence in the separation chamber entry using the imaging setup. The device was created using computer numerical method milling in polymethyl methacrylate (PMMA) with a milling robot (Daltron Neo). The channel was 4 mm wide and 5 mm deep and sample was inserted at the peripheral inlet using a syringe, after which the inlet an outlet channels were closed with an end fitting (red item in Figure 6a).

**Figure 7:**
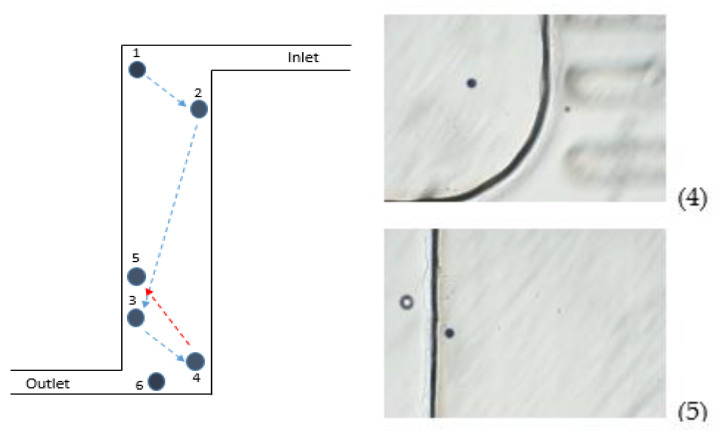
**Left**: Reconstruction of consequent locations of a particle in chamber recorded each after 5 s in a disc rotating at 800 rpm. **Right**: Snapshots of consequent particle locations showing a backwards movement (positions 4 and 5).

The observation of the consequent locations of a 123 μm polystyrene particle (microParticles GmbH) with density 1.05 g/mL, which is close to that of the fluid, 1.07 g/mL, recorded after each 5 s in a disc rotating at 800 rpm (Eppendorf^®^ Centrifuge MiniSpin G), revealed an unusual zig-zag motion pattern and backward motion (Figure 7).

The revealed motion pattern is consistent with the predicted behavior for particles with *ρ_p_* < *ρ_f_*. Indeed, starting from position 1 near the inlet in Figure 7, the particle moves towards the center, and its trajectory deviates to the right, according to Figure 5a, until it reaches the lateral wall (position 2 in Figure 7). At that position, the angle *α* between the radial direction and the lateral wall is smaller than needed for backwards motion, and the particle, after it reaches the wall, drifts along the wall in the direction of the outlet. However, at some point the angle *α* exceeds the critical angle, *α > α_c_*, thus providing the condition for eventual backwards motion and detachment from the wall, and the particle can eventually arrive at the opposite side of the chamber (position 3 in Figure 7). After that, the particle repeats the motion pattern similar to that between positions 1 and 2, and after that it becomes “trapped” at position 4 (Figure 7), where the condition *α > α_c_* holds and, therefore, the particle experiences a strong backwards net force being close to the center of rotation which hits it back to position 5. Finally, after some iterations of back and forth movements, the particle reaches the outlet position.

**Figure 8:**
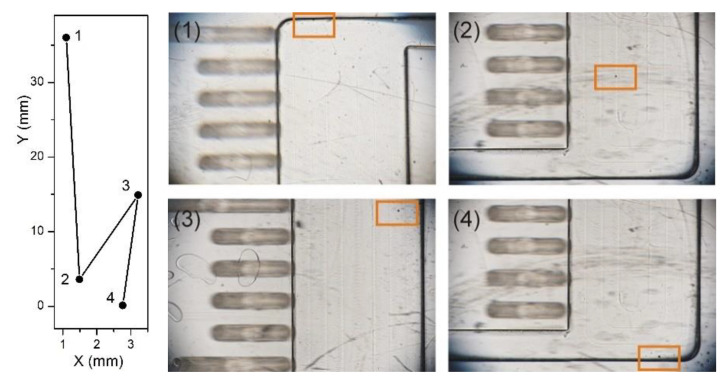
**Left**: Reconstruction of consequent locations of a particle in chamber. **Right**: Snapshots of consequent particle locations (1–4) recorded after 15 s in a disc rotating at 1000 rpm. The pitch of the scale bars is 1 mm.

Similar motion patterns have been revealed in the experiments with 60 μm polystyrene particles, immersed in a solution with density 1.075 g/mL, in a disc rotating at 1000 rpm, when recording particle positions after each 15 s. Figure 8 shows an example of such behavior.

**Figure 9:**
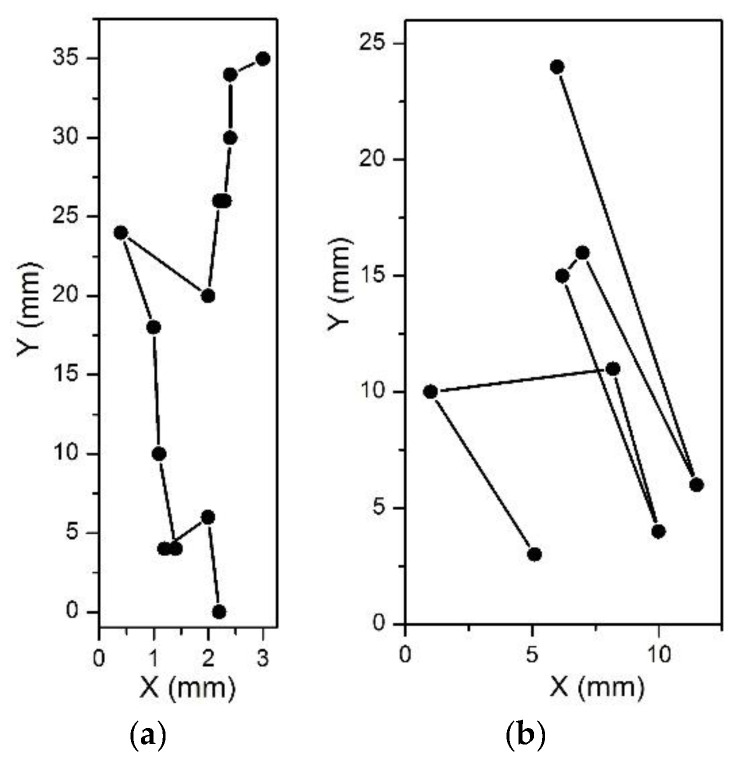
Reconstruction of consequent locations of a particle in chambers with various widths: *w* = 4 mm (**a**) and *w* = 12.5 mm (**b**). (**a**) In the narrow channel, the particle moves towards the center of the rotation following the centrifugal force, with just two backward movements (at *y* = 20 mm and *y* = 4 mm). (**b**) In the wide channel, backwards movements are observed in each step: the particle moves back and forth as predicted in Section 2 (Figure 4 and Figure 5).

Finally, we performed experiments with 123 μm particles in discs with rectangular chambers of various width rotating at 800 rpm and found similar motion patterns (Figure 9). We found that the effect of walls manifests itself more strongly in wide channels, resulting in striking backwards movements. These findings are in qualitative agreement with our theoretical predictions that lateral walls’ misalignment with respect to the radial direction leads to zig-zag and backwards motion patterns. While the above considerations allow for explaining the occurrence of backwards movement, the migration (velocity) is also influenced by physical (mostly reversible but occasionally even irreversible) interactions with the walls during the course of the experiment, which are less straightforward to predict. This will be a subject of a future study.

## 4. Conclusions

The effect of the lateral walls of a centrifuge chamber on the trajectories of moving particles has been analyzed. First, for clarity, we considered massive particles, i.e., with the density higher than that of the solution. In the absence of lateral walls, such a particle moves along a curved trajectory resulting from the interplay of the centrifugal and Coriolis forces, and the distance from the center of rotation to the particle in this case is a monotonic increasing function of time. When placed in a chamber of, e.g., rectangular shape, the trajectories of the particles are impacted by the lateral walls. Depending on the angle between the wall and the radial direction as well as on the rotation speed and on the eventual angular acceleration (that induced additional Euler force exerted on the particle) the particle can move either away or towards the center of rotation. In the former case, the particle, when reaching the wall, drifts along the wall in the same direction as a free particle, i.e., along the centrifugal force. In the latter case, when the relative angle between the wall and the radial direction is large enough and if there is an additional acceleration in the direction of rotation, the particle can move backwards, i.e., opposite to the action of the centrifugal force. This regime can be easily achieved during the initial acceleration of the centrifuge and can essentially contribute to the motion for short centrifugation times. However, while in the case of the forward motion along the wall, the particle can drift all the way towards the outlet, the backwards motion is not as stable. When the direction of motion changes to the opposite, the direction of the inertial Coriolis force also changes to the opposite, and then it acts in the direction of rotation and thus it facilitates detachment of the particle from the wall and moving it in the interior of the chamber. After that, the particle becomes free again and repeats the motion pattern typical for a free particle until it reaches the wall again. This can lead to a rather unusual zig-zag motion pattern with backwards movement parts.

In the case of particles lighter than the solution, i.e., when the particle density is lower than that of the solution, the centrifugation effect forces the particles to move from the periphery of the centrifuge device towards its center. Correspondingly, all the inertial forces change the sign, and the above motion patterns remain but become “inverted” as compared to the above case of particles heavier than the fluid. They look less intuitively clear, but the above analysis allows us to better understand the resulting patterns which were revealed in the experiments with synthetic particles with density slightly lower than the density of the fluid.

The observed effect is in particular important for particles lighter than the solvent, such as eggs. Light particles move towards the center of rotation, where the revealed effect is much stronger than far from the center of rotation. Due to the revealed effect, they can accidentally accumulate near the outlet and thus deteriorate the efficiency of the device. To reduce this undesired effect, the chamber should be properly designed. The obtained results provide a deeper understanding of the centrifugation mechanisms and can be useful for the design optimization of LOD devices.

In terms of importance and potential applications of the presented results:

(*i*) Our work provides an understanding of the effects which were observed and did not have proper explanation (e.g., were attributed to the device imperfections such as surface roughness or eventual leakages, etc.). Our work explicitly revealed the physical mechanisms behind the unusual observed behavior, such as back and forth movements (in particular, when close to the center of rotation). Knowing these mechanisms allows one to better control the motion of particles in LOD devices.
(*ii*) Our findings provide instructions as to how to avoid the undesired effects, e.g., by narrowing the chambers, or by other design improvements. The strength of our approach is that it allows predicting trajectories of particles in any geometry, by a proper description of the boundary conditions, and in this way improve device design to eliminate or to minimize undesired effects.
(*iii*) More challenging, the results can be considered for potential selectivity of particles based on, e.g., their position with respect to the center of rotation, or their individual properties. In this way one can think of collecting various particles separately by a proper device design.

## Supplementary Materials

The following are available online at https://www.mdpi.com/article/10.3390/mi12091032/s1, Figure S1: Trajectory of a particle projected on a narrow (a) and wide (b) rectangular chamber (no parti-cle-wall interaction); particle-wall interaction: the centrifugal, *F_cen_*, Euler, *F_Euler_*, and Coriolis, *F_cor_*, forces and their projections on the lateral wall during the collision of the particle with the wall (c); the resulting velocity in wall contact mode, *v*_1_, and the Coriolis force, *F_cor_*, after the collision with the wall (d): the particle moves in the direction towards the edge, and the Coriolis force is directed towards the wall. The green arrow shows the reaction force exerted on the particle from the wall. Figure S2: Forces and their projections on the lateral wall at the moment of collision with the wall (as in Figure S1 (c)) but for a larger angle between the radius (white dashed line) and the wall (a); the resulting velocity, *v*_1_, and the Coriolis force, *F_cor_*, after the collision with the wall (b): the particle moves in the direction towards the center, and the Coriolis force is directed towards the inner part of the chamber.
