# Supplementary material for: Migration Behavior of Low-Density Particles in Lab-on-a-Disc Devices: Effect of Walls
Source: Micromachines (Basel). 2021 Aug 28;12(9):1032. doi: 10.3390/mi12091032 (PMC8471521; doi:10.3390/mi12091032)
Supplement: Supplementary file 1 [file micromachines-12-01032-s001.zip › micromachines-1330683-SI.pdf]

Supporting Information: Migration Behavior of Low-Density Particles in Lab-on-a-Disc Devices: Effect of Walls

Vyacheslav R. Misko [1], Agata Kryj [1], Aude-Muriel Tamandjo Ngansop [1], Sogol Yazdani [1], Matthieu Briet [1], Namanya Basinda [1,2], Humphrey D. Mazigo [2] and Wim De Malsche [1,*]

1 µFlow Group, Department of Bioengineering Sciences, Vrije Universiteit Brussel, 1050 Brussels, Belgium; veaceslav.misco@vub.be (V.R.M.); Agata.Kryj@vub.be (A.K.); aude-muriel.tamandjo.ngansop@vub.be (A.-M.T.N.); sogol.yazdani@vub.be (S.Y.); matthieu.briet@vub.be (M.B.); namanya.samson.basinda@vub.be (N.B.)

2 Department of Medical Parasitology and Entomology, School of Medicine, Catholic University of Health and Allied Sciences, 33000 Mwanza, Tanzania; humphreymazigo@gmail.com

* Correspondence: Wim.De.Malsche@vub.be; Tel.: +32-2-6293781

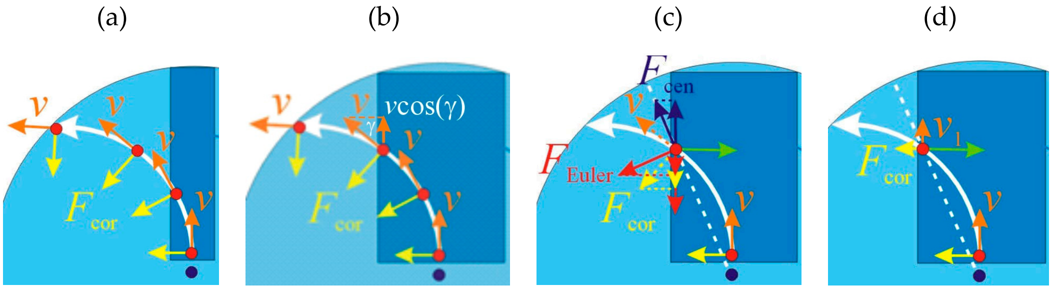

Figure S1. Trajectory of a particle projected on a narrow (**a**) and wide (**b**) rectangular chamber (no particle-wall interaction); particle-wall interaction: the centrifugal, F_{cen}, Euler, F_{Euler}, and Coriolis, F_{cor}, forces and their projections on the lateral wall during the collision of the particle with the wall (**c**); the resulting velocity in wall contact mode, v_1, and the Coriolis force, F_{cor}, after the collision with the wall (**d**): the particle moves in the direction towards the edge, and the Coriolis force is directed towards the wall. The green arrow shows the reaction force exerted on the particle from the wall.

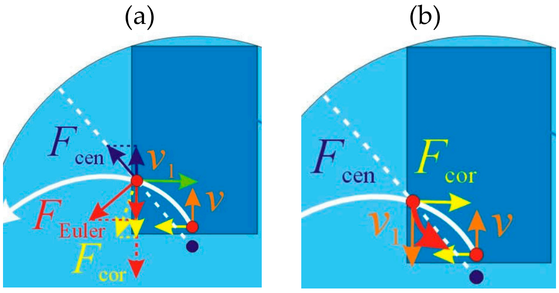

Figure S2. Forces and their projections on the lateral wall at the moment of collision with the wall (as in Figure S1c) but for a larger angle between the radius (white dashed line) and the wall (**a**); the resulting velocity, v_1, and the Coriolis force, F_{cor}, after the collision with the wall (**b**): the particle moves in the direction towards the center, and the Coriolis force is directed towards the inner part of the chamber.
